# Mosquito arbovirus survey in selected areas of Kenya: detection of insect-specific virus

**DOI:** 10.1186/s41182-018-0095-8

**Published:** 2018-06-04

**Authors:** Hanako Iwashita, Yukiko Higa, Kyoko Futami, Peter A. Lutiali, Sammy M. Njenga, Takeshi Nabeshima, Noboru Minakawa

**Affiliations:** 1 Department of Vector Ecology and Environment, Institute of Tropical Medicine, Nagasaki University, 1-12-4 Sakamoto, Nagasaki, 852-8523 Japan; 2 Department of Bacteriology, Graduate School of Medicine, University of the Ryukyus, 207 Uehara, Nishiharacho, Okinawa, 903-0125 Japan; 3 NUITM-KEMRI Project, Kenya Medical Research Institute, Nairobi, Kenya; 4 Eastern and Southern Africa Centre of International Parasite Control (ESACIPAC), Kenya Medical Research Institute, Nairobi, Kenya; 5 Department of Virology, Institute of Tropical Medicine, Nagasaki University, Nagasaki, Japan

**Keywords:** Arbovirus, Insect-specific virus, Culex flavivirus, *Aedes* mosquito, *Culex* mosquito, *Anopheles* mosquito, Busia, Kakamega, Mombasa, Kenya

## Abstract

**Background:**

Many arboviral outbreaks have occurred in various locations in Kenya. Entomological surveys are suitable methods for revealing information about circulating arboviruses before human outbreaks are recognized. Therefore, mosquitoes were collected in Kenya to determine the distribution of arboviruses.

**Methods:**

Various species of mosquitoes were sampled from January to July 2012 using several collection methods. Mosquito homogenates were directly tested by reverse transcription-polymerase chain reaction (RT-PCR) using various arbovirus-targeted primer pairs.

**Results:**

We collected 12,569 mosquitoes. Although no human-related arboviruses were detected, Culex flavivirus (CxFV), an insect-specific arbovirus, was detected in 54 pools of 324 *Culex quinquefasciatus* individuals collected during the rainy season. Of these 54 positive pools, 96.3% (52/54) of the mosquitoes were collected in Busia, on the border of western Kenya and Uganda. The remaining two CxFV-positive pools were collected in Mombasa and Kakamega, far from Busia. Phylogenetic analysis revealed minimal genetic diversity among the CxFVs collected in Mombasa, Kakamega, and Busia, even though these cities are in geographically different regions. Additionally, CxFV was detected in one mosquito pool collected in Mombasa during the dry season. In addition to *Culex* mosquitoes, *Aedes* (*Stegomyia*) and *Anopheles* mosquitoes were also positive for the *Flavivirus* genus. Cell fusing agent virus was detected in one pool of *Aedes aegypti*. Mosquito flavivirus was detected in three pools of *Anopheles gambiae* s.l*.* collected in the dry and rainy seasons.

**Conclusions:**

Although no mosquitoes were positive for human-related arbovirus, insect-specific viruses were detected in various species of mosquitoes. The heterogeneity observed in the number of CxFVs in *Culex* mosquitoes in different locations in Kenya suggests that the abundance of human-related viruses might differ depending on the abundance of insect-specific viruses. We may have underestimated the circulation of any human-related arbovirus in Kenya, and the collection of larger samples may allow for determination of the presence of human-related arboviruses.

## Background

Emergence and re-emergence of vector-borne diseases are crucial public health problems worldwide [1, 2]. In Kenya, many sporadic outbreaks have been reported in geographically different areas [3]. For example, an outbreak of dengue (DEN) fever occurred in the coastal towns of Malindi and Kilifi in 1982 [4], and in 1992–1993, an outbreak of yellow fever (YF) occurred in Rift Valley Province [5]. There were outbreaks of Rift Valley fever (RVF) in 1997 and 2006 [6–8], and an outbreak of chikungunya (CHIK) fever occurred in 2004 in the coastal area of Kenya [9, 10]. In Uganda, an epidemic of o’nyong’nyong (ONN) started in early 1959 and spread to Kenya [11, 12].

In general, febrile diseases caused by viruses are still confused with non-viral diseases, such as malaria [2]. Moreover, cases can remain unnoticed because some arboviral infections are mild and self-limiting during the early stage. Therefore, the number of human arboviral cases might be much higher than has been reported. Even in the absence of clinical outbreaks, historic serosurveys in Kenya can provide important clues about circulating arboviruses in various environments [13]. For instance, Mease et al. in [14] assessed the prevalence of IgG against yellow fever virus (YFV), West Nile virus (WNV), dengue virus (DENV), and chikungunya virus (CHIKV) using serum samples from healthy Kenyans. According to their data, 46.6% of the people in all study areas had antibodies against at least one of these arboviruses [14]. As historic serosurveys in Kenya have documented several arboviruses in geographically different areas [15], a large epidemic of arbovirus can occur anywhere at any time because, as demonstrated recently, many factors such as demographic, geographic environmental and climate change factors can complicate and worsen the situation [16]. Many studies have revealed that a threat of arboviral transmission is present throughout Kenya, regardless of the officially announced reports of outbreaks [17, 18].

Controlling arboviral diseases is difficult because of the complex environment and ecology, including relationships among viruses, vectors, and humans [2, 16, 19]. Multiple vector species are often involved in an arboviral disease, and a single vector can also transmit several diseases. Moreover, primary vectors vary among geographical areas, and the level of vector competence may also vary among species depending on each area [20]. Mosquitoes are known to carry not only human-related viruses but also insect-specific viruses, such as Culex flavivirus and Aedes flavivirus [21]. In addition, interactions between many types of viruses and many other organisms may affect vector competence inside the mosquito [22]. For example, the presence of co-infection with insect-specific virus and WNV has been reported [23]. In this case, co-infection might be considered a factor for the emergence of arbovirus, though the function of insect-specific viruses remains unclear. Assessing the potential for arbovirus outbreaks at the local level can be facilitated by identifying all patterns of relationships, including triangular relationships (human-vector-arbovirus environment), in each area [24]. Moreover, entomological baseline data may contribute to estimations of disease risk and allow precautionary measures to be taken against virus activity. In this study, we mainly selected collection sites where other researchers had previously found or suspected arbovirus activity. For example, border areas are suspected to be areas of potential arbovirus infection because busy transportation hubs may provide many opportunities for human-vector contact [25]. Although the presence of arboviruses has not yet been reported in some indigenous forests in Kenya*,* many species of mosquitoes can serve as bridge vectors of arboviruses, easily spreading sylvatic arboviruses such as sylvatic YF and sylvatic DENV from forests to human environments in these areas in Kenya [26]. We suspected that arboviruses were silently circulating, without outbreak detection. Therefore, an active survey was undertaken in border areas, including coastal boundaries and indigenous forests. The aim of this study was to obtain data regarding the presence of arboviruses in mosquitoes in selected areas of Kenya. Our additional goal was to recognize the main vector species of arboviruses.

## Methods

### Study areas

**Fig. 1:**
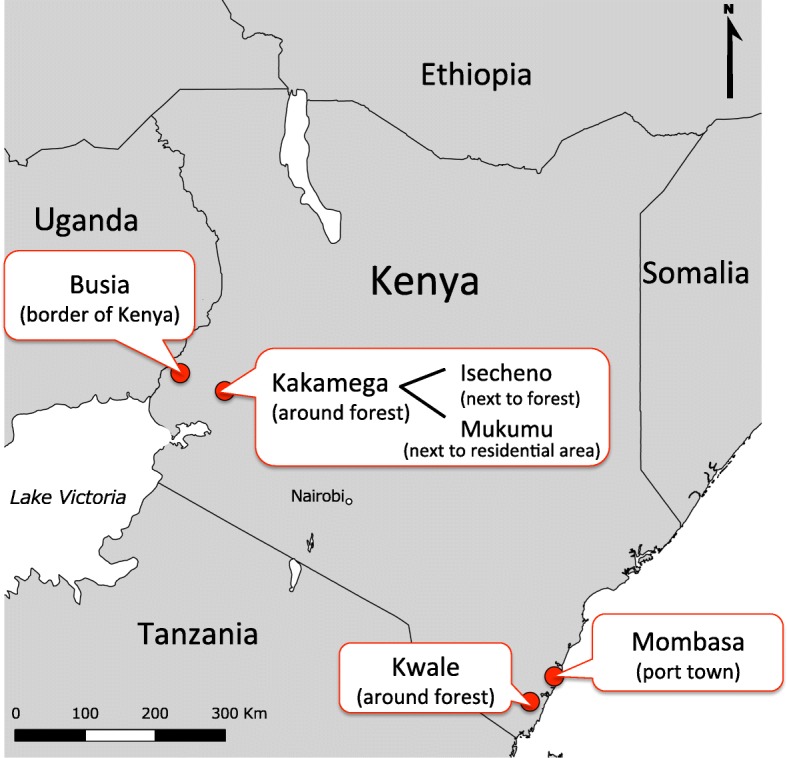
Map of study region. Location of mosquito sampling site in East Kenya; Kwale, Mombasa, and in West Kenya; Busia, Kakamega (Mukumu and Isecheno)

Mosquito sampling was performed in eastern (Mombasa and Kwale) and western (Kakamega and Busia) Kenya, which included a variety of areas, such as urban coastal border, land border, and rural areas next to a forest where there is suspected arbovirus activity (Fig. 1). The sampling was conducted in two different seasons: the rainy season and a season other than the rainy season; March to June in Kenya generally constitutes the rainy season. We initiated this study in January 2012, before the rainy season, which we conventionally termed the dry season. Between January 18 and 26, 2012 (representing dry-season sampling), we conducted a preliminary survey only in eastern Kenya. Between May 9 and June 8, 2012 (representing rainy-season sampling), we conducted the same survey in both eastern and western Kenya.

#### Eastern Kenya: Mombasa (the center: 4^**°**^3.509′S; 39^**°**^40.363′E)

This busy port town includes the urban coastal border with high levels of human activity. Dengue cases have been reported here for approximately the last 30 years [4]. We suspected that due to human activity, arboviral mosquitoes can be easily transported outside this area. Mosquitoes were collected in resident areas in the 2012 dry season (from January 24 to January 26) and in the 2012 rainy season (from May 15 to May 17).

#### Eastern Kenya: Kwale (the center: 4^**°**^10.525′S; 39^**°**^27.087′E)

In this rural area, patches of indigenous forests (Shimba Hills National Reserve) exist next to the residential area. The edge of the indigenous forest can act as a border cross which arboviral mosquitoes can be transported from the forest to the residential area. Mosquitoes were sampled from houses in the 2012 dry season (from January 18 to January 20) and in the 2012 rainy season (from May 9 to May 12).

#### Western Kenya: Kakamega (the center: 0**°**16.923′N; 34**°**45.234′E)

Kakamega forest has a remarkable diversity of insects, birds and animals, which can serve as reservoir hosts of arboviruses [27]. We selected two areas: one exactly next to the indigenous forest (Isecheno), and another, a residential area (Mukumu) along the main road in this region. The edge of the indigenous forest is considered to be a dangerous border of arboviral activity, similar to Kwale. We suspect that the area is easily penetrable by arboviral mosquitoes from forests to residential areas and vice versa. The main road is also regarded as a border, which may encourage transmission of arboviruses. Mosquitoes were collected in Mukumu from June 2 to June 4 and in Isecheno from June 6 to June 8.

#### Western Kenya: Busia (the center: 0^**°**^27.914′N; 34^**°**^5.979′)

Busia is in the western land border (Kenya and Uganda) area, including a busy town with high human activity. Serological surveys were conducted and revealed a high positive rate of antibodies against arboviruses in healthy residents [14]. In this area, many residents may have already suffered from arboviral diseases, with or without symptoms. Transmission between humans and mosquitoes may have been underestimated due to the complicated human activity. Mosquito surveillance can provide other information to show the actual circulation of arboviruses. Mosquitoes were collected from May 25 to May 27.

### Mosquito sampling

In each area, mosquitoes were collected for 3 consecutive days from 13 selected houses within approximately 0.5 km^2^ in each targeted area, except one area (for 4 consecutive days in Kwale in the rainy season). A systematic sampling method was applied for selecting study houses in each targeted area [28]. For example, in Kakamega, the main intersection (0^**°**^16.923′N; 34^**°**^45.234′E) on Kisumu-Kakamega Road was used as the starting point for the systematic sampling of houses. From this point, we established 13 sampling points at 250-m intervals. The nearest house from each point was then selected. The house belongs to a large family (> 5 people), and it was suggested because our study targets human-related arboviruses. Another house was selected if the household head or guardian was not willing to participate in the study. The same method was performed in other areas. Collection methods used the following traps: (i) CDC light traps, (ii) CDC gravid traps, and (iii) BG sentinel traps. Additionally, indoor resting mosquito collection with hand aspirators was performed in all houses. To use 20 traps effectively, we placed 2 types of traps randomly within each of the 13 study houses. We intended to collect as many mosquitoes as possible because arbovirus transmission is usually maintained at a low level in a mosquito population [29]. When the number of mosquitoes collected was insufficient, the position of the traps or type of traps was randomly changed. We used the most effective collection combination with positioning and type of traps at each study site.

In our study, the position of the traps depended on the structure of the house. CDC light traps were suspended > 1.5 m above the ground inside and outside of the houses but not near any other sources of artificial light. CDC gravid traps were placed in a stable area somewhere inside or outside the house where nothing could upset the medium in the pan, for example, under eaves. BG sentinel traps were placed in the house with enough space or outside of the house. CDC light traps were operated from dusk to dawn, whereas other traps were operated for 3–4 days continuously. Resting mosquito collection was performed using oral aspirators by three persons in all rooms of the selected houses in the early morning for 15 min each day; this occurred during all collection periods when the house was visited to remove the mosquito-sampling bags from the traps. To prevent RNA degradation, the captured mosquitoes were kept alive during transfer to the laboratory.

### Mosquito identification

At the laboratory, the collected mosquitoes were killed at − 20 °C and placed on white filter paper in a Petri dish placed on a chill table and identified morphologically to the species level under a stereoscopic microscope using published keys [30–33]. For accurate identification, *Aedes aegypti*, *Culex quinquefasciatus*, *Anopheles funestus*, and *An. rivulorum* were confirmed by polymerase chain reaction (PCR) using specific primers (Table 1).

**Table 1:** Primers used to detect and to sequence arbovirus from mosquito pools in Kenya

Target	Primer name	Nucleotide sequence (5′ to 3′)	Polarity	Product (bp)	Cycle condition	Reference
Universal primers for flavivirus	MAMD	AACATGATGGGRAARAGRGARAA	Forward	252	94°C, 2 min, 1 cycle; 94°C, 1 min, 53°C, 1 min, 72°C, 1 min, 35 cycles; 72°C, 5 min, 1 cycle	Scaramozzino et al. (2001) [48]
	cFD2	GTGTCCCAGCCGGCGGTGTCATCAGC	Reverse			
Universal primers for flavivirus	FLAVI-1	AATGTACGCTGATGACACAGCTGGCTGGGACAC	Forward	854–863	94°C, 5 min, 1 cycle; 94°C, 1 min, 58°C, 1 min, 72°C, 90 s, 45 cycles; 72°C, 10 min, 1 cycle	Ayers et al.(2006) [49]
	FLAVI-2	TCCAGACCTTCAGCATGTCTTCTGTTGTCATCCA	Reverse			
Universal primers for flavivirus (mainly YF)	YF-1	GGTCTCCTCTAACCTCTAG	Forward	675	94°C, 2 min, 1 cycle; 94°C, 30 s, 53°C, 30 s, 72°C, 1 min, 35 cycles; 72°C, 5 min, 1 cycle	Tanaka et al. (1993) [50]
	YF-3	GAGTGGATGACCACGGAAGACATGC	Reverse			
Universal primers for alpha viruses (mainly chikungunya and o’nyong’nyong viruses)	nsP1-S	TAGAGCAGGAAATTGATCC	Forward	354	94°C, 2 min, 1 cycle; 94°C, 30 s, 53°C, 30 s, 72°C, 45 s, 35 cycles; 72°C, 5 min, 1 cycle	Hasebe et al. (2002) [51]
	nsP1-C	CTTTAATCGCCTGGTGGTA	Reverse			
Universal primers for alpha viruses (mainly chikungunya and o’nyong’nyong viruses)	E1-S	TACCCATTCATGTGGGG	Forward	294	94°C, 2 min, 1 cycle; 94°C, 30 s, 53°C, 30 s, 72°C, 45 s, 35 cycles; 72°C, 5 min, 1 cycle	Hasebe et al. (2002) [51]
	E1-C	GCCTTTGTACACCACGAT	Reverse			
Rift Valley virus	RVF009	CCAAATGACTACCAGTCAGC	Forward	400–500	94°C, 2 min, 1 cycle; 94°C, 30 s, 50°C, 30 s, 72°C, 1 min, 35 cycles; 72°C, 5 min, 1 cycle	Jupp et al. (2000) [52] (modified)
	RVF007	GACAAATGAGTCTGGTAGCA	Reverse			
Mosquito RNA marker	Act-2F	ATGGTCGGYATGGGNCAGAAGGACTC	Forward	683	94°C, 2 min, 1 cycle; 94°C, 30 s, 54°C, 30 s, 72°C, 45 s, 35 cycles; 72°C, 5 min, 1 cycle	Staley et al. (2010) [53]
	Act-8R	GATTCCATACCCAGGAAGGADGG	Reverse			
*Culex quinquefaciatus*	ACEpip	GGAAACAACGACGTATGTACT	Forward	610	94°C, 5 min, 1 cycle; 94°C, 30 s, 54°C, 30 s, 72°C, 1 min, 35 cycles; 72°C, 5 min, 1 cycle	Kasai et al. (2008) [39]
	ACEquin	CCTTCTTGAATGGCTGTGGCA	Forward	274		
	B1246s	TGGAGCCTCCTCTTCACGG	Reverse			
*Aedes aegypti*	18SFHIN	GTAAGCTTCCTTTGTACACACCGCCCGT	Forward	550	97°C, 4 min, 1 cycle; 96°C, 30 s, 48°C, 30 s, 72°C, 2 min, 30 cycles; 72°C, 4 min, 1 cycle	Higa et al. (2010) [54]
	aeg.r1	TAACGGACACCGTTCTAGGCCCT	Reverse			
*Anopheles funestus, Anopheles rivulorum*	UV	TGTGAACTGCAGGACACAT	Forward		94°C, 2 min, 1 cycle; 94°C, 30 s, 45°C, 30 s, 72°C, 40 s, 30 cycles; 72°C, 5 min, 1 cycle	Koekemoer et al. (2002) [55]
	FUN	GCATCGATGGGTTAATCATG	Reverse	505		
	RIV	CAAGCCGTTCGACCCTGATT	Reverse	411		

Note: Each 25 μl reaction mixture contained. (Accupower TM PCR PreMix kit with 2 μl template, 15.2 μl sterile water, and 1.4 μl of 100 pmol/μl each of primers)

### Mosquito processing

A maximum of 30 individuals were pooled according to species, sex, physiological status, (i.e., unfed, blood fed, or gravid), and collection site and then were frozen in liquid nitrogen. For virus detection, we used all pools collected during the dry season, with each category (male, unfed, fed and gravid) examined separately. In contrast, for the pools collected during the rainy season, only unfed and gravid mosquito pools of *Ae. aegypti*, *An. funestus*, *An. gambiae s.l.*, and *Cx. quinquefasciatus* were used. Moreover, both unfed and gravid mosquitoes were combined for some pools of each species. Blood-fed mosquitoes were excluded to prevent contamination of the virus contained in a blood meal, though we did utilize blood-fed mosquitoes collected during the dry season because of the small sample size. For the samples collected during the rainy season, we concentrated on detecting viruses in only female pools, excluding those that were blood-fed.

Pooled specimens were placed in a 1.5-ml microcentrifuge tube with 300 μl of minimal essential medium (MEM) (minimum essential medium containing 10% foetal bovine serum, L-glutamine, penicillin, streptomycin, and amphotericin B). The mosquitoes were ground in MEM, and the homogenate was centrifuged; 200 μl of the supernatant was collected and kept at − 80 °C for future use (for cell culture). To maintain approximately 100 μl of the suspension, 75 μl of lysis buffer was added. The homogenates were prepared using sterile, RNase-free utensils.

### Total RNA extraction and virus identification by reverse transcription-PCR

Total RNA was extracted from each pool of mosquitoes using an extraction kit (SV Total RNA Isolation System, Promega, Tokyo, Japan) according to the manufacturer’s instructions. RNA was eluted in 50 μl of sterile distilled water. Reverse transcription reactions were performed to synthesize first-strand cDNA using RNA to cDNA EcoDry Premix (Random Hexamers) (Clontech Laboratories, Inc., Mountain View, CA, USA). The cDNA was amplified by PCR using an AccuPower^™^ PCR Premix Kit (Bioneer Co., Daejon, Korea) with virus-specific primers (Table 1), and the products were evaluated by 1.5% agarose gel electrophoresis. For all positive samples, products of the expected size were extracted from the gel and were purified using a MonoFas DNA Purification Kit (GL Sciences, Tokyo, Japan). Purified amplicons were bidirectionally sequenced using a BigDye Terminator version 3.1 Cycle Sequencing Kit (Applied Biosystems, Foster City, CA, USA) and analyzed with an ABI3130 Genetic Analyzer (Applied Biosystems). Nucleic acid sequences were compared with those in the GenBank database using the BLAST program.

The process was repeated for three universal primers for flavivirus (the main targets are DENV, YFV, and WNV), two universal primers for alpha viruses (the main targets are ONN virus and CHIKV) and single primer sets for RVFV (phlebovirus) (Table 1). For flaviviruses and alpha viruses, we prepared multiple primer sets to detect not only a well-known virus but also novel viruses. In the case of flavivirus detection, all pools were initially screened for flavivirus RNA by using universal flavivirus primer sets cFD2 and MAMD, which target the non-structural protein 5 (NS5) gene. To identify human-related flaviviruses, such as DENV, YFV, and WNV, all pools were screened with primer sets YF-1 and YF-3. To generate a larger NS5 cDNA segment for sequencing, putative positive samples detected using previous primer sets (cFD2 and MAMD) were again screened for flavivirus RNA using another universal flavivirus primer set (FLAVI1 and FLAVI2) targeting the NS5 gene. Confirmed bands of approximately 860 bp were sequenced as described above. In the case of alpha virus detection, primer sets (nsP1-S and nsP1-C; E1-S and E1-C) designed based on the genes non-structural protein 1 (nsP1) and glycoprotein E1 (E1) were used for amplification.

The following inactivated viruses available in the laboratory were used as positive controls: DEN-1 (Hawaii strain), YFV (17D strain, attenuated live vaccine strain), WNV (NY99 strain), CHIKV (S27 strain, African prototype), RVFV (Smithburn strain, attenuated live vaccine strain) (All positive controls were kindly provided by Dr. S Inoue). As a quality control for the detection step, each cDNA was checked by PCR using the mosquito β-actin primer.

### Calculation of infection rates

We calculated the minimum infection rate (MIR) of arboviruses in each mosquito species at each site using the Poolscreen2 program [34]. MIR is expressed as the number of pools infected per 1000 mosquitoes tested, and it assumes that only one mosquito is positive in a pool. To determine the number of flavivirus-positive samples, the results using primer sets cFD2 and MAMD were employed. MIR was calculated when at least 100 mosquitoes were tested per species per site.

### Phylogenetic analysis

For virus species identification, the collected sequences were confirmed by an alignment search in gene databases using MEGA6 with the ClustalW method [35]. Phylogenetic and molecular evolutionary analyses were conducted by using the p-distance option with the neighbor-joining (NJ) method. Bootstrap analyses were performed with 1000 replicates. Representative flavivirus sequences were used in the phylogenetic analysis as outgroup sequences.

## Results

### Mosquito collection

#### During the dry season in eastern Kenya (Table 2)

In Kwale (January 18–20, 2012), we employed a cumulative number of 39 trap sessions (per day per house) in 13 houses for 3 days (total numbers of each trap session per day per house were 12 BG sentinel, 15 CDC light, and 12 CDC gravid trap sessions) and a cumulative number of 39 aspirator catch sessions (per day per house) in 13 houses for 3 days using a 3-person aspirator catch team in each house. We collected 179 mosquitoes in the following subset of attempts: 3 BG sentinel trap sessions, 10 CDC light trap sessions, 6 CDC gravid trap sessions, and 7 aspirator catches. In Mombasa (January 24–26, 2012), we collected 872 mosquitoes by the same cumulative number of trap sessions as in Kwale. The collection methods entailed 7 BG sentinel trap sessions, 14 CDC light trap sessions, 12 CDC gravid trap sessions, and 33 aspirator catches. The total number of mosquitoes collected in Kwale and Mombasa was 1051. Of these mosquitoes, 796 (75.7%) were identified as females. For these samples collected during the dry season, all species were tested, including males of each species (70 pools) (Table 2). Only five mosquitoes were not identified and were excluded.

**Table 2:** Summary of mosquitoes collected in the dry season in East Kenya

Study site		Kwale			Mombasa		
Collection methods employed (number of trap sessions)^#^		As; 39, BG; 12, CDC; 15, GT; 12			As; 39, BG; 12, CDC; 15, GT; 12		
Methods collected mosquitoes (number of trap sessions)^#^		As; 7, BG; 3, CDC; 10, GT; 6			As; 33, BG; 7, CDC; 14, GT; 12		
Collection period		January 18–20, 2012 (3 days)			January 24–26, 2012 (3 days)		
Number of houses		13 houses			13 houses		
Species	Physiological status	No. collected	Pools	Positive pool	No. collected	Pools	Positive pool
*Ae. aegypti*	Fed				2	1	0
	Unfed	3	3	0	16	2	0
*An. coustani*	Fed	1	1	0			
*An. funestus*	Fed	1	1	0			
*An. gambiae s.l.*	Fed	3	1	0			
	Unfed	8	1	1	2	1	1
*An. longipulpis*	Fed	1	1	0			
*An. rivulorum*	Fed	4	1	0			
*Anopheles* sp.	Male	1	1	0			
*Cx. cinereus*	Gravid	1	1	0			
*Cx. decens*	Unfed	2	1	0			
	Gravid	4	1	0			
*Cx. quinquefasciatus*	Male	15	1	0	235	10	1
	Fed	30	2	0	105	6	0
	Unfed	19	1	0	375	13	0
	Gravid	64	4	0	129	7	0
*Cx. laticinctus*	Gravid	5	1	0			
*Cx. simpsoni*	Male	1	1	0			
	Unfed				2	1	0
*Cx. univiittetus*	Unfed				2	1	0
*Culex* sp.	Male				1	1	0
	Unfed	1	1	0	3	1	0
*Mansonia* sp.	Fed	2	1	0			
	Unfed	8	1	0			
Others	Male	2					
	Unfed	3					
Total		179	26	1	872	44	2

^#^Abbreviations of collection methods are *As* aspirator, *BG*: *BG* sentinel trap, *CDC*:CDC light trap, *GT* CDC gravid trap

#### During the rainy season in eastern and western Kenya (Table 3)

In Kwale (May 9–12, 2012), we employed a cumulative number of 57 trap sessions (per day per house) in 13 houses for 4 days (total numbers of each trap session per day per house were 30 BG sentinel, 16 CDC light, and 22 CDC gravid trap sessions) and a cumulative number of 48 aspirator catches (per day per house) in 13 houses for 4 days using a 3-person aspirator catch team in each house. We collected 2592 mosquitoes in the following subset of attempts: 25 BG sentinel trap sessions, 11 CDC light trap sessions, 22 CDC gravid trap sessions, and 42 aspirator catches. In Mombasa (May 15–17, 2012), we employed a cumulative number of 42 trap sessions (per day per house) in 13 houses for 3 days (total numbers of trap sessions were 13 BG sentinel traps, 12 CDC light traps, and 17 CDC gravid traps) and a cumulative number of 30 aspirator catch sessions (per day per house) in 13 houses for 3 days using a 3-person aspirator catch team in each house. We collected 1974 mosquitoes in the following subset of attempts: 12 BG sentinel trap sessions, 11 CDC light trap sessions, 17 CDC gravid trap sessions, and 28 aspirator catches. In Busia (May 25–27, 2012), we employed a cumulative number of 45 trap sessions (per day per house) in 13 houses for 3 days (total numbers of trap sessions were 18 BG sentinel, 12 CDC light, and 15 CDC gravid trap sessions) and a cumulative number of 36 aspirator catch sessions (per day per house) in 13 houses for 3 days using 3-person aspirator catch team in each house. We collected 4598 mosquitoes in the following subset of attempts: 17 BG sentinel trap sessions, 12 CDC light trap sessions, 15 CDC gravid trap sessions, and 36 aspirator catches. In Kakamega (Mukumu) (June 2–4, 2012), we employed a cumulative number of 51 trap sessions (per day per house) in 13 houses for 3 days (total numbers of each trap sessions per day per house were 15 BG sentinel, 18 CDC light, and 18 CDC gravid trap sessions) and a cumulative number of 39 aspirator catches (per day per house) in 13 houses for 3 days using 3-person aspirator catch team in each house. We collected 2087 mosquitoes in the following subset of attempts: 13 BG sentinel trap sessions, 16 CDC light trap sessions, 15 CDC gravid trap sessions, and 34 aspirator catches. In Kakamega (Isecheno) (June 6–8, 2012), we employed a cumulative number of 57 trap sessions (per day per house) in 13 houses for 3 days (total numbers of trap sessions per day per house were 15 BG sentinel, 21 CDC light, and 21 CDC gravid trap sessions) and a cumulative number of 39 aspirator catch sessions (per day per house) in 13 houses for 3 days using a 3-person aspirator catch team in each house. We collected 267 mosquitoes in the following subset of attempts: 8 BG sentinel trap sessions, 11 CDC light trap sessions, 17 CDC gravid trap sessions, and 20 aspirator catches.

**Table 3:** Summary of mosquitoes collected in the rainy season in East and West Kenya

Study area		East Kenya			East Kenya			West Kenya			West Kenya			West Kenya		
Study site		Kwale			Mombasa			Busia			Kakamega (Mukumu)			Kakamega (Isecheno)		
Collection methods employed (number of trap sessions)^#^		As; 48, BG; 30, CDC; 16, GT; 22			As; 30, BG; 13, CDC; 12, GT; 17			As; 36, BG; 18, CDC; 12, GT; 15			As; 39, BG; 15, CDC; 18, GT; 18			As; 39, BG; 15, CDC; 21, GT; 21		
Methods collected mosquitoes (number of trap sessions)^#^		As; 42, BG; 25, CDC; 11, GT; 22			As; 28, BG; 12, CDC; 11, GT; 17			As; 36, BG; 17, CDC; 12, GT; 15			As; 34, BG; 13, CDC; 16, GT; 15			As; 20, BG; 8, CDC; 11, GT; 17		
Collection period (days)		May 9–12, 2012 (4 days)			May 15–17, 2012 (3 days)			May 25–27, 2012 (3 days)			June 2–4, 2012 (3 days)			June 6–8, 2012 (3 days)		
Number of houses		13 houses			13 houses			13 houses			13 houses			13 houses		
Species	Physiological status	No. collected	Pool	Positive pool	No. collected	Pool	Positive pool	No. collected	Pool	Positive pool	No. collected	Pool	Positive pool	No. collected	Pool	Positive pool
*Aedes* sp.	Male				2											
	Unfed	3			2			1						1		
*Ae. aegypti*	Male				2											
	Unfed	11	8	0	49	14	1^*^	4	8	0	3	3	0	2	2	0
	Gravid	2			7			6			1			1		
*An. brumripes*	Unfed	1												2		
*An. funestus*	Male							3								
	Fed							5								
	Unfed							59	7	0						
	Gravid							2								
*An. gambiae s.l.*	Male							22								
	Fed	2						37						3		
	Unfed	18	3	0				402	34	1	3	4	0	3	3	0
	Gravid							9			1					
*An. garnhami*	Fed										1					
	Unfed	6														
*An. parensis*	Unfed	1														
*An. rivulorum*	Unfed							4	3	0						
	Gravid										1	1	0			
*Anopheles* sp.	Male	1														
	Fed	2														
	Unfed	6						2			1					
	Gravid													1		
*Cx. decens*	Male													1		
	Gravid													1		
*Cx .quinquefasciatus*	Male	355			540			1554			332			38		
	Fed	202			351			331			840			79		
	Unfed	106	106	0	443	52	1^*^	1243	113	52^***^	472	46	1^**^	53	7	0
	Gravid	1867			554			906			431			79		
*Cx. simpsoni*	Unfed							1								
*Cx. univiittetus*	Unfed							1								
*Culex* sp.	Male				3											
	Fed	3			1											
	Unfed	4			2						1			0		
	Gravid	1			1									3		
Lutzia	Gravid							2								
Others	Male				2											
	Unfed	1			15			3								
	Gravid							1								
Total		2592	117	0	1974	66	2	4598	165	53	2087	54	1	267	12	0

Note: We used only unfed and gravid mosquitoes for the pools to detect arboviruses. Unfed and gravid mosquitoes were separated into each category, but some of them were combined into one pool

^*^The pool comprised only gravid mosquitoes

^**^The pool comprised only unfed mosquitoes

***Pools consisted of unfed and gravid mosquitoes

^#^Abbreviations of “collection methods” are *As* aspirator, *BG* BG sentinel trap, *CDC* CDC light trap, *GT* CDC gravid trap

In total, we collected 11,518 mosquitoes at all sampling sites. Of these mosquitoes collected during the rainy season, 8663 (75.2%) were identified as female. Only unfed and gravid female mosquitoes (414 pools) were used for virus detection in samples collected during the rainy season (Table 4). The number of mosquitoes collected in Kakamega (Isecheno) was one order of magnitude lower than that collected at the other study sites.

**Table 4.** Information of positive samples for insect specific arbovirus

Places	Season	Species of mosquito	No. of mosquitoes	Physiological status of used pools	No. pools	No. positive pools	MIR^*^	MIR Lower-upper limits	Physiological status of positive pool
*Ae. aegypti* mosquito pools									
Kwale	Dry	*Ae. aegypti*	3	Unfed	3	0	NA	NA	
Kwale	Rain	*Ae. aegypti*	13	Unfed, gravid	8	0	NA	NA	
Mombasa	Dry	*Ae. aegypti*	18	Fed, unfed	3	0	NA	NA	
Mombasa	Rain	*Ae. aegypti*	56	Unfed, gravid (♂; excluded)	14	1	NA	NA	Female, gravid
Busia	Rain	*Ae. aegypti*	10	Unfed, gravid	8	0	NA	NA	
Kakamega (Mukumu)	Rain	*Ae. aegypti*	4	Unfed, gravid	3	0	NA	NA	
Kakamega (Isecheno)	Rain	*Ae. aegypti*	3	Unfed, gravid	2	0	NA	NA	
*Cx. quinquefasciatus* mosquito pools									
Kwale	Dry	*Cx. quinquefasciatus*	128	♂, fed, unfed, gravid	8	0	NA	NA	
Kwale	Rain	*Cx. quinquefasciatus*	1973	Unfed, gravid (♂, fed; excluded)	106	0	NA	NA	
Mombasa	Dry	*Cx. quinquefasciatus*	844	♂, fed, unfed, gravid	36	1	1.18	0.07–5.75	♂
Mombasa	Rain	*Cx. quinquefasciatus*	997	Unfed, gravid (♂, fed; excluded)	52	1	1.01	0.06–4.89	Female, gravid
Busia	Rain	*Cx. quinquefasciatus*	2149	Unfed, gravid (♂, fed; excluded)	113	52	32.26	24.42–42.12	Female, unfed + gravid
Kakamega (Mukumu)	Rain	*Cx. quinquefasciatus*	903	Unfed, gravid (♂, fed; excluded)	46	1	1.11	0.06–5.37	Female, unfed
Kakamega (Isecheno)	Rain	*Cx. quinquefasciatus*	132	Unfed, gravid (♂, fed; excluded)	7	0	NA	NA	
*An. gambiae* mosquito pools									
Kwale	Dry	*An. gambiae*	11	Fed, unfed	2	1	NA	NA	Female, unfed
Kwale	Rain	*An. gambiae*	18	Unfed, (fed; excluded)	3	0	NA	NA	
Mombasa	Dry	*An. gambiae*	2	Unfed	1	1	NA	NA	Female, unfed
Mombasa	Rain	*An. gambiae*	0		0	0	NA	NA	
Busia	Rain	*An. gambiae*	411	Unfed, gravid (♂, fed; excluded)	34	1	2.44	0.14–11.87	Female, unfed
Kakamega (Mukumu)	Rain	*An. gambiae*	4	Unfed, gravid	4	0	NA	NA	
Kakamega (Isecheno)	Rain	*An. gambiae*	3	Unfed (fed; excluded)	3	0	NA	NA	

^*^Minimum infection rate

### Arbovirus detection

Overall, 484 pools consisting of 7788 mosquitoes were tested. The selected species collected in both seasons for the detection of arbovirus were *Ae. aegypti* (41 pools), *An. funestus* (8 pools), *An. gambiae* s.l. (47 pools), *An. rivulorum* (5 pools), and *Cx. quinquefasciatus* (368 pools). The following species of mosquitoes collected during only the dry season from East Kenya were also used for detection: *An. coustani* (1 pool), *An. longipalpis* (1 pool), *Cx. cinereus* (1 pool), *Cx. decens* (2 pools), *Cx. laticinctus* (1 pool), *Cx. simpsoni* (2 pool), *Cx. univittatus* (1 pool), *Anopheles* sp. (1 pool), *Culex* sp. (3 pools) and *Mansonia* sp. (2 pools). Although we collected 2 individuals of *Cx. decens* (1 male and 1 female from Isecheno), *Cx. simpsoni* (1 female from Busia), and *Cx. univittatus* (1 female from Busia) during the rainy season, we did not use these specimens for detection because of their small sample numbers compared to all other pools during the rainy season.

### Human-related arboviruses from all mosquitoes

All pools were negative for human-related arboviruses, such as DENV, YFV, WNV, ONN, and CHINV.

### Mosquito-related arboviruses from *Culex quinquefasciatus*

Using the primer sets cFD2 and MAMD, PCR bands were observed for 54 female *Cx. quinquefasciatus* pools during the rainy season and 1 male *Cx. quinquefasciatus* pool during the dry season in Mombasa (Tables 2 and 3). The nucleotide sequences for positive PCR reactions amplified using the primer sets cFD2 and MAMD from all these pools were compared with the GenBank database (BLAST), and sequencing results of all samples were 99% identical to the homologous region of Culex flavivirus (CxFV) strain Uganda08 (GQ165808.1). When we limited our analysis to female mosquitoes only, Busia yielded the most positive pools (52 pools) followed by Bamburi (1 pool) and Mukumu (1 pool).

**Fig. 2:**
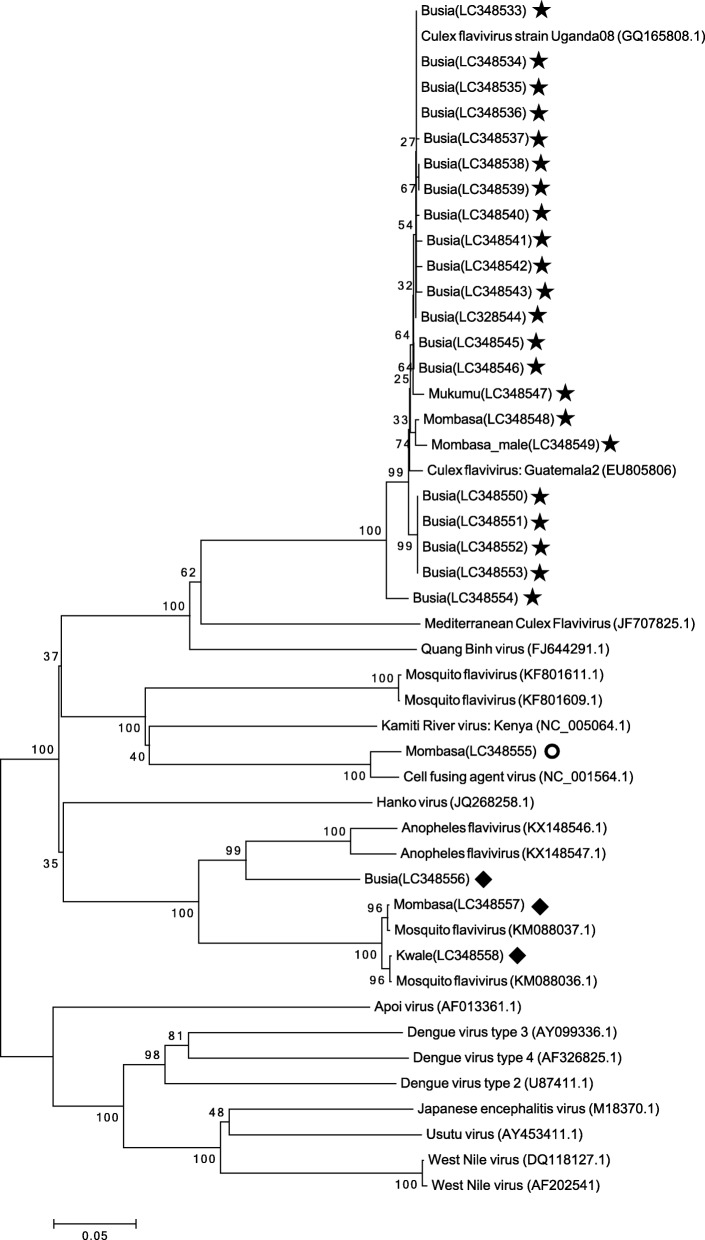
Phylogenetic tree of the positive sequences based on the 597 nucleotides of the NS5 gene. The tree was constructed by employing the program MEGA 6, using the neighbor-joining method and distance-p model with 1000 bootstrap replicates. GenBank accession numbers are indicated in the parenthesis in the tree. Numbers on internal branches indicate bootstrap values for 1000 replicates. Our samples are marked with star (*Cx. quinquefasciatus*), with circle (*Ae. aegypti*), and with diamond shape (*An. gambiae*)

To generate a larger NS5 cDNA segment for sequencing to be used in phylogenetic analyses, only pools that were positive for flavivirus using the primer sets cFD2 and MAMD were amplified with the primer sets FLAVI1 and FLAVI2. Bands of approximately 860 nt (597 nt was used) were observed, and nucleotide sequencing was successful for 22 pools of *Cx. quinquefasciatus* (21 female pools and 1 male pool) among 55 pools (54 female pools and 1 male pool). The genomic sequences obtained using both primer sets (FLAVI1 and FLAVI2) share similar nucleotide sequence identity (99%) with CxFV from Uganda (GenBank: GQ165808.1). This result was the same as that using the primer sets cFD2 and MAMD. A phylogenetic tree was constructed with the NJ method using NS5 gene sequences of 22 CxFV strains by adding CxFV NS5 gene sequences from Uganda (GenBank: GQ165808.1) and Guatemala (GenBank: EU805806) obtained from BLAST. Additionally, NS5 gene sequences of human-related flaviviruses, such as WNV (GenBank: DQ118127.1, GenBank: AF202541), DNV (GenBank: AY099336.1, GenBank: AF326825.1, GenBank: U87411.1), and Japanese encephalitis virus (GenBank: M18370.1), were included as outgroup sequences. The NS5 gene sequences of our samples from Kenya clustered with CxFV NS5 gene sequences from Uganda and Guatemala. Although Busia, Kakamega, and Mombasa are in completely different regions of Kenya, the phylogenetic tree shows sequence similarity (Fig. 2).

### Mosquito-related arboviruses from *Ae. aegypti* and *An. gambiae*

The PCR products using the primer sets FLAVI1 and FLAVI2 for one pool of *Ae. aegypti* were shown to correspond to cell-fusing agent virus (CFAV) (NC_001564.1, 96% BLAST identity). In terms of *An. gambiae* s.l. pools, PCR products using the same primer sets as above were observed for three female pools, consisting of one pool from Kwale and one pool from Mombasa (both collected during the dry season) and one pool from Busia (collected during the rainy season). The nucleotide sequencing results of the two samples collected in Kwale and Mombasa were similar to mosquito flavivirus sequences (KM088036.1 and KM088037.1, 99% BLAST identity) reported from Kenya. The sequence of the sample collected from one pool from Busia was moderately divergent from the other two, being most similar a sequence of Anopheles flavivirus (KX148546.1, 85% BLAST identity) reported from Liberia. According to Kuno et al., a viral species is defined as the same group of viruses with > 84% nucleotide sequence identity among them [36]. Our sequence analysis demonstrated slightly higher nucleotide sequence identity than this cut-off. Therefore, the viruses from *An. gambiae* s.l. collected in Busia represent a variant of the closely related Anopheles flavivirus. The phylogenetic analyses including arboviruses from *Cx. quinquefasciatus* are presented in Fig. 2.

### Minimum infection rate (MIR)

Although our study sites were geographically limited, MIR for *Cx. quinquefasciatus* showed a heterogeneous distribution for this species among the selected sites. Busia was the region with the highest MIR among all *Cx. quinquefasciatus* pools collected in Kenya (Table 4). Other *Cx. quinquefasciatus* pools revealed only one positive pool, with an MIR of approximately 1.0 (Table 4). Furthermore, taking into account differences in sampling efficiency among the study sites, seasons and traps, the *Cx. quinquefasciatus* specimens collected in Busia showed a higher MIR (MIR = 32.26; 95% CI = 24.42–42.12) than those collected in Mombasa during the rainy season (MIR = 1.01; 95% CI = 0.06–4.89) and during the dry season (MIR = 1.18; 95% CI = 0.07–5.75), and those collected in Kakamega during the rainy season (MIR = 1.11; 95% CI = 0.06–5.37). CxFV was detected in Mombasa during the dry season in a male pool as well as in female pools; however, there were no positive samples found in female pools during the dry season. No differences in MIR were found between the dry and rainy seasons in Mombasa, even though the pools of male and fed mosquitoes collected in the rainy season were not tested. Because of the limited number of samples, it is uncertain whether heterogeneity exists among *Ae. aegypti* and *An. gambiae* MIRs.

## Discussion

In this study in Kenya, we did not detect any human-related arboviruses, and the main vector species of arboviruses were not found. Instead, we did detect mosquito-specific arboviruses from many types of mosquitoes. In particular, high prevalence of CxFV is *Cx. quinquefasciatus* was found in Busia, and this strain of CxFV is similar to one reported in Uganda by Cook et al. [37, 38]. Additionally, a similar CxFV was detected in each female pool from Mombasa and Kakamega. These areas in Kenya are separated by great distances. Additional sampling in the area between Busia and Kakamega in western Kenya and in the area between Kakamega and Mombasa in middle to eastern Kenya will likely increase the precision of the data regarding CxFV prevalence and geographic variation in Kenya. At present, the consequences of this geographic variation in Kenya are not clear. Moreover, we detected CxFV in one male pool collected in Mombasa. This result suggests that vertical maintenance may be common, even though Mombasa is an area with a lower positive rate compared to Busia.

Although many studies have reported mosquito-specific flavivirus detection in *Culex* and *Aedes* [39], there is little information about flaviviruses from anopheline mosquitoes, except for a few recent reports from Africa [40, 41]. In addition to *Ae. aegypti*, we also obtained flavivirus sequences from *An. gambiae* s.l. Our phylogenetic data using flavivirus NS5 gene sequences suggest that the sequences from *Ae. aegypti* are related to CFAV and that the sequences from *An. gambiae* s.l. are most closely related to mosquito flaviviruses (KM088037.1 and KM088036.1) from *An. gambiae* s.l. in West Africa and Kenya [40, 41]. Overall, reports of mosquito-specific flaviviruses are increasing.

Our results are based on partial sequences (NS5) of flaviviruses directly detected in mosquitoes. However, other regions of flavivirus nucleotide sequences (such as a region of NS3) were not determined, and there is a possibility that these sequences differ. Thus, further sequence information might be required, especially for a novel mosquito flavivirus, to establish the detailed taxonomic status of arboviruses. None of the mosquitoes in our samples were infected with human-related flaviviruses, though the detection rate might have been slightly higher if we had performed cell culture. Another limitation is the small sample size, and the number of mosquito species was also small. Larger studies are needed to provide a more accurate view of the prevalence of arboviruses.

Additionally, the abundance of *Ae. aegypti*, one of the most effective arboviral vectors in the human environment, obtained was relatively smaller than we expected*.* This mosquito is thought to have originated from Africa and to have been introduced to other continents such as Asia and South America through maritime trade [1]. Because this mosquito can easily adapt to urban areas on these continents, DENV transmitted by *Ae. aegypti* has become a major threat to humans. In this study, there were no positive pools of arboviruses, including DEN and CHIK, among 107 female *Ae.* aegypti samples. It is clear that this small sample size is insufficient. Additionally, due to this small sample size, the existence of another important vector, *Aedes albopictus*, cannot be determined, even though the distribution of this Asian-based mosquito has already been extended throughout the world, including West and Central Africa [42, 43]. Currently, this mosquito is not reported in Kenya. However, methods of collecting *Aedes* mosquitoes in Kenya remain an issue. We recognize that the effectiveness of the BG sentinel trap is quite low in certain areas such as Africa [44], though we did not analyze the effectiveness of each trap.

Here, we report the detection of CxFV from *Cx. quinquefasciatus*, CFAV from *Ae. aegypti*, mosquito flavivirus from *An. gambiae* s.l., and a new virus from *An. gambiae* s.l*.* However, we did not detect any arboviruses that are responsible for human disease. Many individuals might be exposed to a considerable risk of arbovirus infection in Kenya. Muyeku et al. reported the seroprevalence of CHIKV, YFV, and WNV in children at a hospital in Busia. According to their data for 2010, the virus with the highest positive rate was WNV (31% of 296 tested) followed by YFV (17% of 310 tested) and CHIV (11% of 298 tested) [45]. Moreover, there is an anecdotal report that the WNV infection rate might be higher than that reported because many infections are not obvious or are mild among those who live on the border of Kenya and Uganda, where this virus was first isolated in 1937 [46]. Regardless, the detection of human-related arboviruses in mosquitoes is very difficult in the absence of an outbreak. Our results, which indicate relatively high CxFV positivity among *Cx. quinquefasciatus* mosquitoes in Busia, might support risk prediction for future patterns of epidemics of arboviral infection. One previous study reported a positive association between insect-specific flaviviruses and human-related arboviruses, such as WNV [23]. Interestingly, Bolling et al. [47] identified early suppression of WNV infection in *Culex pipiens* naturally infected with CxFV. This suppression is one of the possible explanations for the lack of arbovirus detection, despite the high prevalence of CxFV in *Cx. quinquefasciatus* in our study. Thus, it is important to determine whether mosquitoes infected with mosquito-specific flaviviruses are resistant or susceptible to infection with other human-related flaviviruses. Future research on these viruses and their potential interactions with other flaviviruses in arthropod vectors will provide important new insight into not only virological but also public health aspects.

## Conclusions

Insect-specific viruses were detected in various species of mosquitoes. In particular, the abundance of CxFV in *Culex* mosquitoes in Busia is higher than in other areas of Kenya. We suspect that this heterogeneity in various areas of Kenya may reflect the heterogeneity of the abundance of human-related virus vectors. These results, together with the absence of positive pools of human-related arbovirus, can be used as a baseline for future studies of human arboviruses. Future efforts to detect the circulation of arboviruses will help clarify the relationship between human-related arboviruses and various arboviruses, including insect-specific viruses. Detection methods that are more sensitive, such as next-generation sequencing (NGS), will facilitate obtaining real data about the presence of arboviruses.

## Abbreviations

CFAV: Cell-fusing agent virus
CHIKV: Chikungunya virus
CxFV: Culex flavivirus
DEN: Dengue
DENV: Dengue virus
MIR: Minimum infection rate
NJ: Neighbor-joining
ONN: O’nyong’nyong
PCR: Polymerase chain reaction
RVF: Rift Valley fever
WNV: West Nile virus
YF: Yellow fever
YFV: Yellow fever virus

## References

[1] Gubler DJ (2002). The global emergence/resurgence of arboviral diseases as public health problems. Arch Med Res.

[2] Weaver SC, Reisen WK (2010). Present and future arboviral threats. Antivir Res.

[3] Sutherland LJ, Cash AA, Huang YJ, Sang RC, Malhotra I, Moormann AM (2011). Serologic evidence of arboviral infections among humans in Kenya. Am J Trop Med Hyg.

[4] Johnson BK, Ocheng D, Gichogo A, Okiro M, Libondo D, Kinyanjui P (1982). Epidemic dengue fever caused by dengue type 2 virus in Kenya: preliminary results of human virological and serological studies. East Afr Med J.

[5] Sanders EJ, Marfin AA, Tukei PM, Kuria G, Ademba G, Agata NN (1998). First recorded outbreak of yellow fever in Kenya, 1992-1993. I. Epidemiologic investigations. Am J Trop Med Hyg.

[6] Nguku PM, Sharif S, Mutonga D, Amwayi S, Omolo J, Mohammed O (2010). An investigation of a major outbreak of Rift Valley fever in Kenya: 2006–2007. Am J Trop Med Hyg.

[7] Woods CW, Karpati AM, Grein T, McCarthy N, Gaturuku P, Muchiri E (2002). An outbreak of Rift Valley fever in Northeastern Kenya, 1997-98. Emerg Infect Dis.

[8] Crabtree M, Sang R, Lutomiah J, Richardson J, Miller B (2009). Arbovirus surveillance of mosquitoes collected at sites of active Rift Valley fever virus transmission: Kenya, 2006–2007. J Med Entomol.

[9] Sergon K, Njuguna C, Kalani R, Ofula V, Onyango C, Konongoi LS (2008). Seroprevalence of chikungunya virus (CHIKV) infection on Lamu Island, Kenya, October 2004. Am J Trop Med Hyg.

[10] Charrel RN, De Lamballerie X, Raoult D (2007). Chikungunya outbreaks - the globalization of vectorborne diseases. N Engl J Med.

[11] Haddow AJ, Davies CW, Walter AJ (1960). O’nyong-nyong fever: an epidemic virus disease in East Africa - introduction. Trans R Soc TropMed Hyg.

[12] Williams MC, Woodall JP, Corbet PS, Gillett JD (1965). O’nyong-nyong fever: an epidemic virus disease in East Africa. 8. Virus isolations from anopheles mosquitoes. Trans R Soc Trop Med Hyg.

[13] Geser A, Henderson BE, Christensen S (1970). A multipurpose serological survey in Kenya. 2. Results of arbovirus serological tests. Bull World Health Organ.

[14] Mease LE, Coldren RL, Musila LA, Prosser T, Ogolla F, Ofula VO (2011). Seroprevalence and distribution of arboviral infections among rural Kenyan adults: a cross-sectional study. Virol J.

[15] Sang RC, Dunster LM (2001). The growing threat of arbovirus transmission and outbreaks in Kenya: a review. East Afr Med J.

[16] Weaver SC, Barrett AD (2004). Transmission cycles, host range, evolution and emergence of arboviral disease. Nat Rev Microbiol.

[17] Lutomiah J, Bast J, Clark J, Richardson J, Yalwala S, Oullo D (2013). Abundance, diversity, and distribution of mosquito vectors in selected ecological regions of Kenya: public health implications. J Vector Ecol.

[18] Labeaud AD, Sutherland LJ, Muiruri S, Muchiri EM, Gray LR, Zimmerman PA (2011). Arbovirus prevalence in mosquitoes, kenya. Emerg Infect Dis.

[19] Gould EA, Higgs S (2009). Impact of climate change and other factors on emerging arbovirus diseases. Trans R Soc Trop Med Hyg.

[20] Vazeille M, Moutailler S, Coudrier D, Rousseaux C, Khun H, Huerre M (2007). Two chikungunya isolates from the outbreak of La Reunion (Indian Ocean) exhibit different patterns of infection in the mosquito, *Aedes albopictus*. PLoS One.

[21] Hoshino K, Isawa H, Tsuda Y, Sawabe K, Kobayashi M (2009). Isolation and characterization of a new insect flavivirus from Aedes albopictus and Aedes flavopictus mosquitoes in Japan. Virology.

[22] Ciota AT, Kramer LD (2013). Vector-virus interactions and transmission dynamics of West Nile virus. Viruses.

[23] Newman CM, Cerutti F, Anderson TK, Hamer GL, Walker ED, Kitron UD (2011). Culex Flavivirus and west Nile virus mosquito coinfection and positive ecological association in Chicago, United States. Vector Borne Zoonotic Dis.

[24] Gu W, Novak RJ (2004). Short report: detection probability of arbovirus infection in mosquito populations. Am J Trop Med Hyg.

[25] Weaver SC (2013). Urbanization and geographic expansion of zoonotic arboviral diseases: mechanisms and potential strategies for prevention. Trends Microbiol.

[26] Hanley KA, Monath TP, Weaver SC, Rossi SL, Richman RL, Vasilakis N (2013). Fever *versus* fever: the role of host and vector susceptibility and interspecific competition in shaping the current and future distributions of the sylvatic cycles of dengue virus and yellow fever virus. Infect Genet Evol.

[27] Berens DG, Farwig N, Schaab G, Boehning-Gaese K (2008). Exotic guavas are foci of forest regeneration in Kenyan farmland. Biotropica.

[28] Southwood TRE, ECOLOGICAL METHODS second Edition 1977.

[29] Gu W, Unnasch TR, Katholi CR, Lampman R, Novak RJ (2008). Fundamental issues in mosquito surveillance for arboviral transmission. Trans R Soc Trop Med Hyg.

[30] Harbach RE (1985). Pictorial keys to the genera of mosquitoes, subgenera of *Culex* and the species of *Culex* (*Culex*) occurring in southwestern Asia and Egypt, with a note on the subgeneric placement of *Culex deserticola* (Diptera: Culicidae). Mosq Syst.

[31] Huang YM (2002). A pictorial key to the mosquito genera of the world, including subgenera of Aedes and Ochlerotatus (Diptera: Culicidae). Ins Koreana.

[32] Reinert JF (1999). Descriptions of Zavortinkius, a new subgenus of Aedes, and the eleven included species from the Afrotropical region (Diptera: Culicidae).

[33] Rueda LM (2004). Pictorial keys for the identification of mosquitoes (Diptera:Culicidae) associated with dengue virus transmission. Zootaxa.

[34] Biggerstaff BJ (2006). PooledInfRate, version 3.0: a Microsoft excel add-in to compute prevalence estimates from pooled samples.

[35] Tamura K, Dudley J, Nei M, Kumar S (2007). MEGA4: molecular evolutionary genetics analysis (MEGA) software version 4.0. Mol Biol Evol.

[36] Kuno G, Chang GJ, Tsuchiya KR, Karabatsos N, Cropp CB (1998). Phylogeny of the genus Flavivirus. J Virol.

[37] Cook S, Moureau G, Harbach RE, Mukwaya L, Goodger K, Ssenfuka F (2009). Isolation of a novel species of flavivirus and a new strain of Culex flavivirus (Flaviviridae) from a natural mosquito population in Uganda. J Gen Virol.

[38] Mwangangi JM, Midega J, Kahindi S, Njoroge L, Nzovu J, Githure J (2012). Mosquito species abundance and diversity in Malindi, Kenya and their potential implication in pathogen transmission. Parasitol Res.

[39] Kasai S, Komagata O, Tomita T, Sawabe K, Tsuda Y, Kurahashi H (2008). PCR-based identification of Culex pipiens complex collected in Japan. Jpn J Infect Dis.

[40] Fauver JR, Grubaugh ND, Krajacich BJ, Weger-Lucarelli J, Lakin SM, Fakoli LS (2016). West African Anopheles gambiae mosquitoes harbor a taxonomically diverse virome including new insect-specific flaviviruses, mononegaviruses, and totiviruses. Virology.

[41] Villinger J, Mbaya MK, Ouso D, Kipanga PN, Lutomiah J, Masiga DK (2017). Arbovirus and insect-specific virus discovery in Kenya by novel six genera multiplex high resolution melting analysis. Mol Ecol Resour.

[42] Paupy C, Delatte H, Bagny L, Corbel V, Fontenille D (2009). Aedes albopictus, an arbovirus vector: from the darkness to the light. Microbes Infect.

[43] Kraemer MU, Sinka ME, Duda KA, Mylne AQ, Shearer FM, Barker CM (2015). The global distribution of the arbovirus vectors Aedes aegypti and Ae. albopictus. elife.

[44] Sivagnaname N, Gunasekaran K (2012). Need for an efficient adult trap for the surveillance of dengue vectors. Indian J Med Res.

[45] Muyeku MI, Seroprevalence of chikungunya, yellow fever and West Nile viruses in children at the Alupe District Hospital in Western Kenya. http://erepository.uonbi.ac.ke/bitstream/handle/11295/3785/Muyeku_Seroprevalence%20of%20Chikungunya%2c%20Yellow%20fever%20and%20West%20Nile%20Viruses%20in%20Children.pdf?sequence=1&isAllowed=y (2011). Accessed 5 Dec 2017.

[46] Smithburn KC, Hughes TP, Burke AW, Paul JH (1940). A neurotropic virus isolated from the blood of a native of Uganda. Am J Trop Med Hyg.

[47] Bolling BG, Olea-Popelka FJ, Eisen L, Moore CG, Blair CD (2012). Transmission dynamics of an insect-specific flavivirus in a naturally infected Culex pipiens laboratory colony and effects of co-infection on vector competence for West Nile virus. Virology.

[48] Scaramozzino N, Crance JM, Jouan A, DeBriel DA, Stoll F, Garin D (2001). Comparison of flavivirus universal primer pairs and development of a rapid, highly sensitive heminested reverse transcription–PCR assay for detection of flaviviruses targeted to a conserved region of the NS5 gene sequences. J Clin Microbiol.

[49] Ayers M, Adachi D, Johnson G, Andonova M, Drebot M, Tellier R (2006). A single tube RT-PCR assay for the detection of mosquito-borne flaviviruses. J Virol Methods.

[50] Tanaka M (1993). Rapid identification of flavivirus using the polymerase chain reaction. J Virol Methods.

[51] Hasebe F, Parquet MC, Pandey BD, Mathenge EG, Morita K, Balasubramaniam V (2002). Combined detection and genotyping of chikungunya virus by a specific reverse transcription-polymerase chain reaction. J Med Virol.

[52] Jupp PG, Grobbelaar AA, Leman PA, Kemp A, Dunton RF, Burkot TR (2000). Experimental detection of Rift Valley fever by reverse transcription–polymerase chain reaction assay in large samples of mosquitoes. J Med Entomol.

[53] Staley M, Dorman KS, Bartholomay LC, Fernández-Salas I, Farfan-Ale JA, Loroño-Pino MA (2010). Universal primers for the amplification and sequence analysis of actin-1 from diverse mosquito species. J Am Mosq Control Assoc.

[54] Higa Y, Toma T, Tsuda Y, Miyagi IA (2010). Multiplex PCR-based molecular identification of five morphologically related, medically important subgenus *Stegomyia* mosquitoes from the genus *Aedes*(Diptera: Culicidae) found in the Ryukyu Archipelagon Japan. Jpn J Infect Dis.

[55] Koekemoer LL, Kamau L, Hunt RH, Coetzee M (2002). Cocktail polymerase chain reaction assay to identify members of the *Anopheles funestus* (Diptera: Culicidae) group. Am J Trop Med Hyg.

